# Radiation Rescue: Mesenchymal Stromal Cells Protect from Lethal Irradiation

**DOI:** 10.1371/journal.pone.0014486

**Published:** 2011-01-05

**Authors:** Claudia Lange, Bärbel Brunswig-Spickenheier, Heike Cappallo-Obermann, Katharina Eggert, Ursula M. Gehling, Cornelia Rudolph, Brigitte Schlegelberger, Kerstin Cornils, Jozef Zustin, Andrej-Nikolai Spiess, Axel R. Zander

**Affiliations:** 1 Clinic for Stem Cell Transplantation, Department of Cell and Gene Therapy, University Medical Center Hamburg-Eppendorf, Hamburg, Germany; 2 Department of Andrology, University Medical Center Hamburg-Eppendorf, Hamburg, Germany; 3 Department of Hepatobiliary and Transplant Surgery, University Medical Center Hamburg-Eppendorf, Hamburg, Germany; 4 Institute of Cell and Molecular Pathology, Hannover Medical School, Hannover, Germany; 5 Institute for Osteopathology, University Medical Center Hamburg-Eppendorf, Hamburg, Germany; National Cancer Institute, United States of America

## Abstract

**Background:**

Successful treatment of acute radiation syndromes relies on immediate supportive care. In patients with limited hematopoietic recovery potential, hematopoietic stem cell (HSC) transplantation is the only curative treatment option. Because of time consuming donor search and uncertain outcome we propose MSC treatment as an alternative treatment for severely radiation-affected individuals.

**Methods and Findings:**

Mouse mesenchymal stromal cells (mMSCs) were expanded from bone marrow, retrovirally labeled with eGFP (bulk cultures) and cloned. Bulk and five selected clonal mMSCs populations were characterized in vitro for their multilineage differentiation potential and phenotype showing no contamination with hematopoietic cells. Lethally irradiated recipients were i.v. transplanted with bulk or clonal mMSCs. We found a long-term survival of recipients with fast hematopoietic recovery after the transplantation of MSCs exclusively without support by HSCs. Quantitative PCR based chimerism analysis detected eGFP-positive donor cells in peripheral blood immediately after injection and in lungs within 24 hours. However, no donor cells in any investigated tissue remained long-term. Despite the rapidly disappearing donor cells, microarray and quantitative RT-PCR gene expression analysis in the bone marrow of MSC-transplanted animals displayed enhanced regenerative features characterized by (i) decreased proinflammatory, ECM formation and adhesion properties and (ii) boosted anti-inflammation, detoxification, cell cycle and anti-oxidative stress control as compared to HSC-transplanted animals.

**Conclusions:**

Our data revealed that systemically administered MSCs provoke a protective mechanism counteracting the inflammatory events and also supporting detoxification and stress management after radiation exposure. Further our results suggest that MSCs, their release of trophic factors and their HSC-niche modulating activity rescue endogenous hematopoiesis thereby serving as fast and effective first-line treatment to combat radiation-induced hematopoietic failure.

## Introduction

The management of patients suffering from acute radiation syndromes (ARS) still remains a major challenge. Survival of radiation induced bone marrow failure depends on the dose of radiation received and the intensity of supportive care which can protect from otherwise lethal infection and give surviving stem cells a chance to expand. To provide the best possible care for radiation accident victims in acts of terrorism or catastrophic incidences, medical countermeasures need to be made within the first few days for optimal efficacy [1]. The “response category concept” proposed by Fliedner et al [2] evaluates the radiation induced tissue damage and rates the hematopoietic score 4-H4 (the highest score for hematopoietic damage) as case with little probability for autologous recovery. Combined approaches including presenting symptoms, biomarkers and physical dosimetry are employed to categorize affected individuals for best medical countermeasures [3].

Overall measures include supportive care, treatment with growth factors within the first two weeks after radiation exposure, or hematopoietic stem cell transplantation (HSCT). Since radiation effects on blood stem cells occur at doses generally lower than on other critical organs, the rapidly emerging changes in the peripheral blood cell lineages dictate the treatment options. Animal and human studies indicate that hematopoietic pluripotent stem cells have a D_0_ of about 95cG as indicated by Fliedner et al [1]. D_0_ is the dose increment that reduces the cell survival to 37%. In fact, total body exposure at doses more than 7–8 Gy total body irradiation (TBI) in human corresponds to medullar eradication. Under this threshold spontaneous recovery from residual hematopoietic stem and progenitor cells may be expected within 30–50 days but going through cytopenic phases of granulocytic, megakaryocytic and erythrocytic lineages. HSCT should be considered if the victim's HSC pool is essentially irreversibly damaged. Interestingly, even after TBI, intrinsically radioresistant stem cells have been detected in distinct bone marrow (BM) areas comprising a residual hematopoietic stem and progenitor cell pool [4]. ARS does not only imply damage to the bone marrow. In a dose-dependent matter, it can also emerge as gastrointestinal and cerebrovascular syndromes leading to development of multiple organ dysfunctions [4]. Damage to the whole organism is related to a systemic inflammatory response. Different target organs are affected due to activation of the innate immune system, resulting in a significant release of inflammatory cytokines [5]. The pathophysiology appears comparable to that of acute graft-versus-host disease (GvHD) following allogeneic stem cell transplantation where a similar “cytokine storm” has been observed [6]. Long-term effects of ionizing radiation have been well documented in atomic bomb survivors in whom persistent signs of inflammation, e.g. increased plasma levels of tumor necrosis factor-α (TNF−-α), interferon-β, interleukin-6, and C-reactive protein, have been reported [7]. Additionally, oxidative stress after high dose ionizing radiation has been involved in delayed morbidity [4]. Management of ARS therefore relies on tissue damage repair processes that might be supported by therapies directed at mitigation of inflammation [4].

Efforts to improve outcome for affected individuals focus on the stem cell niche. Therefore, visionary therapies should augment niche activity to accelerate hematopoietic recovery in vivo. Several studies have demonstrated that BM osteoblasts regulate the HSC pool size in vivo via the Jagged1-Notch signaling pathway [1]. For example, parathyroid hormone receptor activation can increase the number of osteoblastic cells, resulting in Notch1-mediated expansion of HSCs [8]. One integrative part of the BM stroma are the mesenchymal stromal cells (MSCs), also described as osteoblastic progenitors [9]. MSCs have been proven to intervene with acute organ impairments. Cotransplanted with HSCs, MSCs augment hematopoietic recovery after chemo- or radiotherapy significantly decreasing the time to full hematopoietic and particularly platelet reconstitution [10]. Additionally, there is a large body of evidence for MSCs effectiveness in the treatment of steroid resistant GvHD without any side effects even when obtained from BM of third-party donors [11], [12]. No HLA-matching is needed between donor and recipient because MSCs have been shown to be hypoimmunogenic and are not recognized by the recipients immune system even after repeated injections [11], [12]. Finally, MSCs secrete a plethora of bioactive molecules [13], [14]. Among these, several essential hematopoietic growth factors including IL6, IL11, LIF, SCF, and Flt3 ligand are produced but also factors with immunomodulatory effects, e.g. TGF-β1, prostaglandin E2, indoleamine 2,3-dioxygenase, and others. [14], [15]. Additionally, vascular endothelial growth factor (VEGF) secreted by MSCs in large amounts might interfere with an early apoptotic cell death after irradiation [4]. Therefore, MSCs might be a good candidate for the modulation of hematopoietic niche activity.

Altogether, we assumed that MSCs with their comprehensive trophic potential could serve as a readily available treatment option after severe radiation exposure.

## Results

### MSCs promote hematopoietic recovery after lethal irradiation

**Figure 1:**
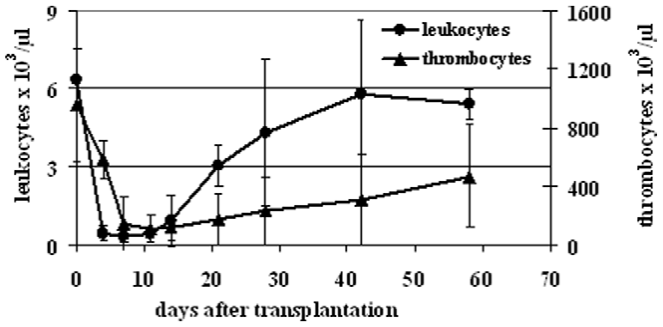
Mouse MSCs rescue mice after total body irradiation. Transplantation of bulk mMSCs led to a normalization of the peripheral white blood cell count within 4 weeks. Thrombocyte recovery needed approx. 8 weeks for normalization. Thus, results are comparable to blood recovery after HSC transplantation.

Dynamic evaluation of peripheral blood counts of animals treated with bulk MSCs revealed similar leukocyte and thrombocyte recovery as observed in recipients transplanted with HSCs [16] reaching normalization of white blood cells after 4 weeks (Figure 1). Seven months after transplantation, 2/3 of recipients still were alive (Table 1) and hematologically well with a normal distribution of peripheral blood cell (PB) populations (Table 2).

**Table 1:** Phenotypical characterization of mMSCs and recipients' survival rates after transplantation.

	CD34	CD45	CD59	CD90	CD105	CD117	Sca-1	survival at 7 months [%]
bulk	1.6	0.5	95.4	0.5	85.9	0.9	96.7	19/28 [67.9]
IXH8	***9.8***	***4.1***	97.4	2.7	***1.6***	1.5	99.2	15/17 [88.2]
IVH7	1.2	1.3	54.7	0.5	94.1	2.8	81.9	2/12 [16.7]
IXC2	0.9	2.2	79.6	1.2	94.0	1.5	90.2	3/10 [30]
VIIIE7	1.2	1.1	71.1	2.0	93.1	1.5	96.4	4/10 [40]
VF10	2.2	2.2	45.9	0.7	74.0	3.4	77.9	3/10 [30]
radiation control	nd	nd	nd	nd	nd	nd	nd	0/15 [0]

Cultures of eGFP-transduced bulk and cloned mMSC after extended expansion were positive for CD59, CD105 and Sca-1 but negative for the hematopoietic markers CD34, CD45, CD117 and for CD90 by flow cytometry. Clone IXH8 was different from all other cultures in its expression of CD34/CD45 and negativity of CD105 (shown in bold italic). Transplantation with this clone resulted in the highest survival rate of the irradiated recipients, suggesting elevated CD34 and CD45 and no CD105 expressions might be a prerequisite of the high rescue capability. nd, not done.

**Table 2:** Peripheral blood cell populations in mMSC transplanted animals.

lymphocytes	neutrophils	monocytes	eosinophils
72%±3	21%±3	5%±2	2%±1

The distribution of white blood cells 5 months after bulk mMSC transplantation was counted using Pappenheim-stained blood smears.

**Figure 2:**
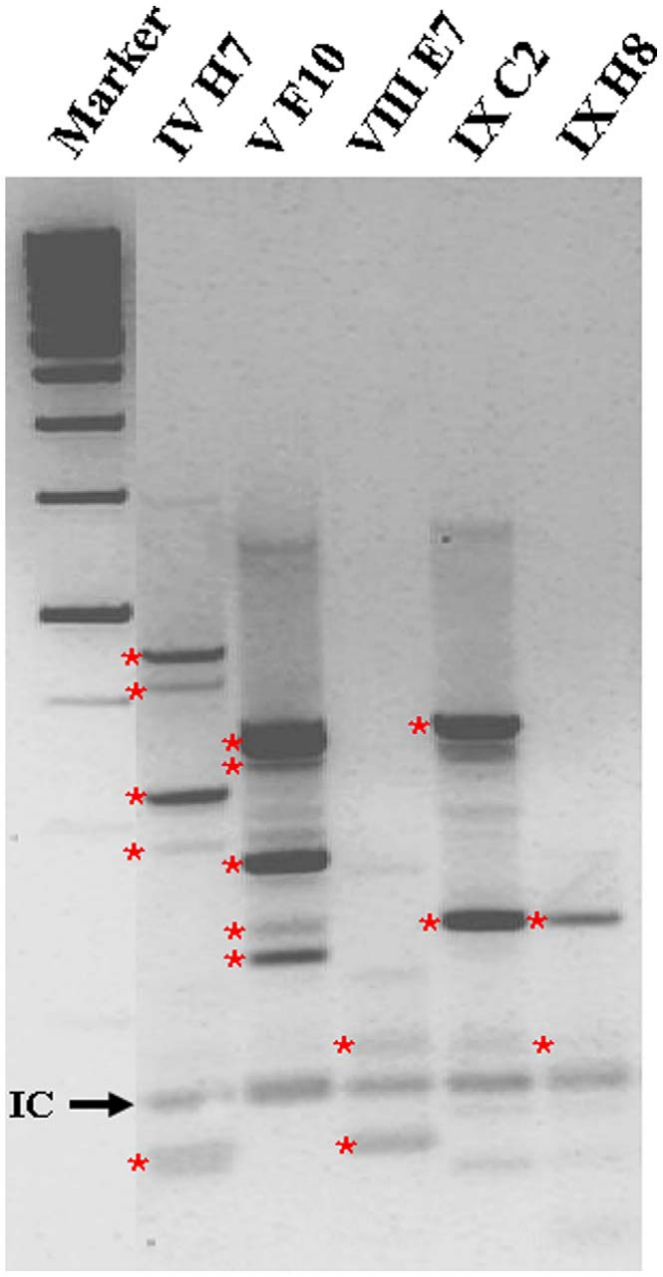
Integration site pattern of clonal mMSCs. The clonal mMSCs were investigated using LM-PCR. Each of the clones represents a specific pattern of integration sites. Bands marked with red asterix were subjected to sequencing for further characterization (see Table S1). IC, internal control.

Using ligation-mediated (LM-) PCR we identified specific integration site (IS) patterns for each single clone in vitro (Figure 2). For mMSC clone IVH7 we identified 2 IS, for VF10 3 IS, for VIIIE7 1 IS, for IXC2 2 IS and for IXH8 1IS. Integration sites shown twice might be due to incomplete digestion of the genomic DNA. The thickness of the bands doesn't resemble minor or major integration sites but is inherent to the method. Further analysis of single integration sites is shown in Table S1.

**Figure 3:**
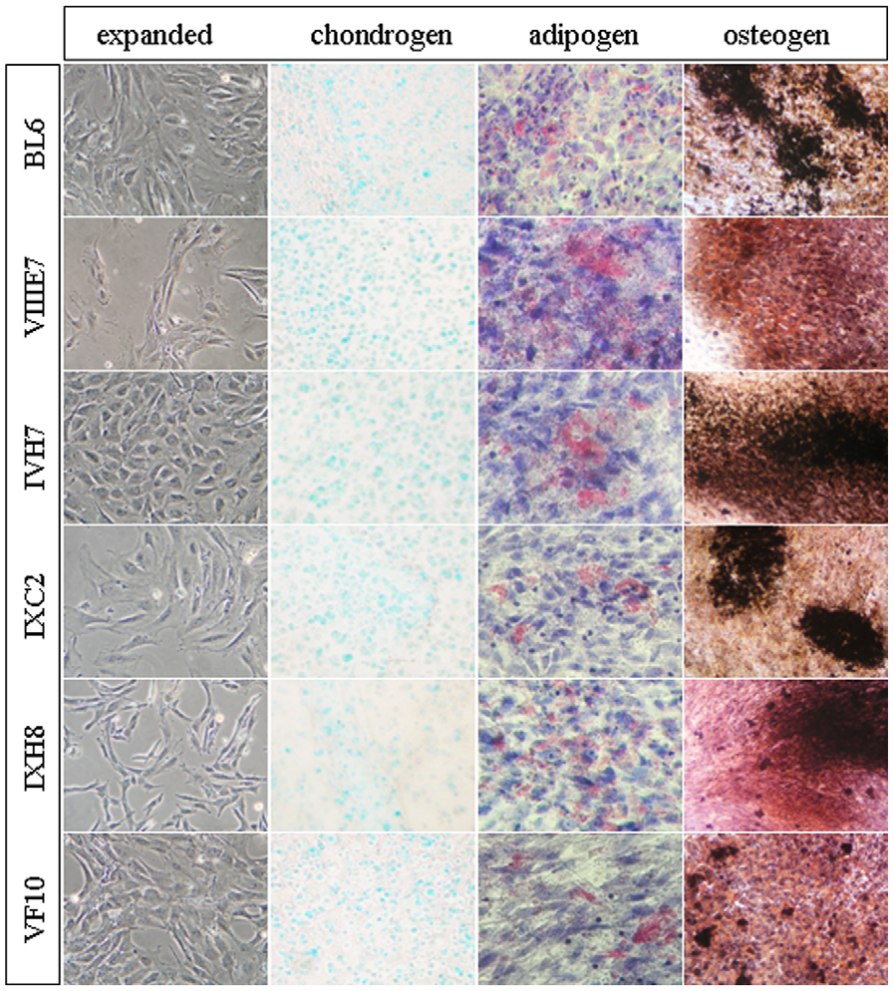
Differentiation potential of mMSCs. Mouse MSCs were generated from male C57BL/6J bone marrow and expanded for 9 passages. Expanded mMSC were retrovirally transduced with eGFP (bulk) and cloned. Five clones with sufficient growth were selected and further expanded. They differed regarding their morphology and growth pace. Cells from passages 14–16 were induced to differentiate into adipogenic, osteogenic and chondrogenic cells. All clones and the parental bulk cells demonstrated three-lineage differentiation capability. Noninduced controls were negative for the respective stainings (not shown).

Transplantation of clonal mMSCs resulted in a superior survival rate of recipients treated with clone IXH8 (88%) compared to survival rates of approx. 30% obtained with other clones (Table 1). No control animals without cell transplantation survived the TBI longer than 3 weeks. Impressively, clone IXH8 morphologically was different from all other cultures (Figure 3) without any flattened cells and additionally showed a distinct phenotype with increased CD34 and CD45 but no CD105 expression. All other characteristics of this clone corresponded to the ISCT criteria [17] (Figure 3, Table 1), questioning the relevance of CD105 expression for MSC characterization.

### Transplanted donor cells are detectable short- but not long-term

**Figure 4:**
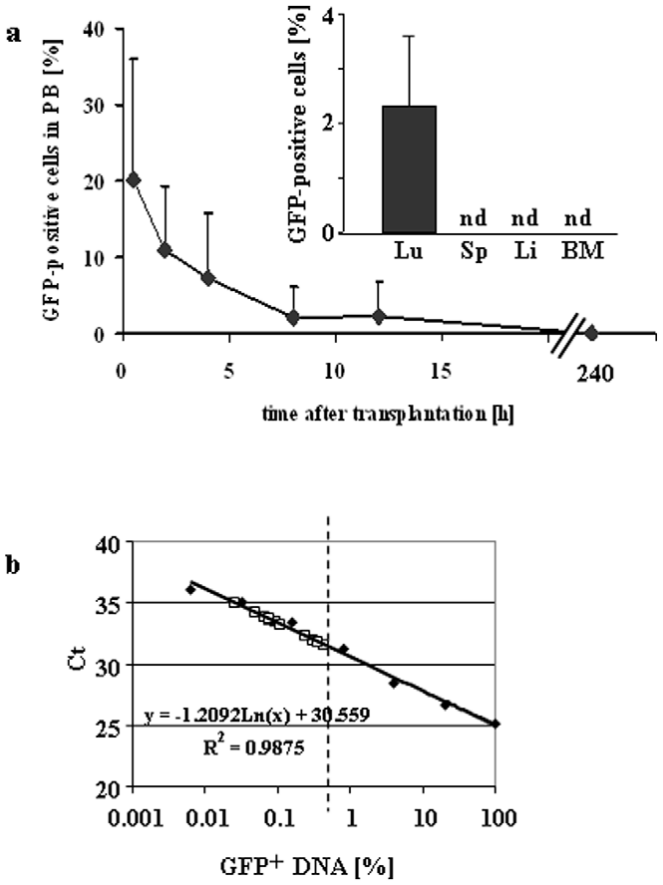
Donor mMSCs are not detectable long-term. (a) Tracking of eGFP-labeled clonal IXH8 donor mMSCs after transplantation revealed a fast decrease in PB. Within 8 hours, approx. 2% were quantified in PB and none after 10 days (n = 8 for each time point). Insert: mMSCs accumulated in lungs (Lu) within 24 h and disappeared within 10 days (not shown). Spleen (Sp), liver (Li) and BM were negative at d1. (b) Based on standard dilutions (filled symbols), no eGFP signals (open symbols) above the assay's detection limit of approx. 0.5% were detected in the BM of long-term survivors reconstituted with mMSCs of clone IXH8 (dashed line, detection limit of qPCR). nd, not detected.

**Figure 5:**
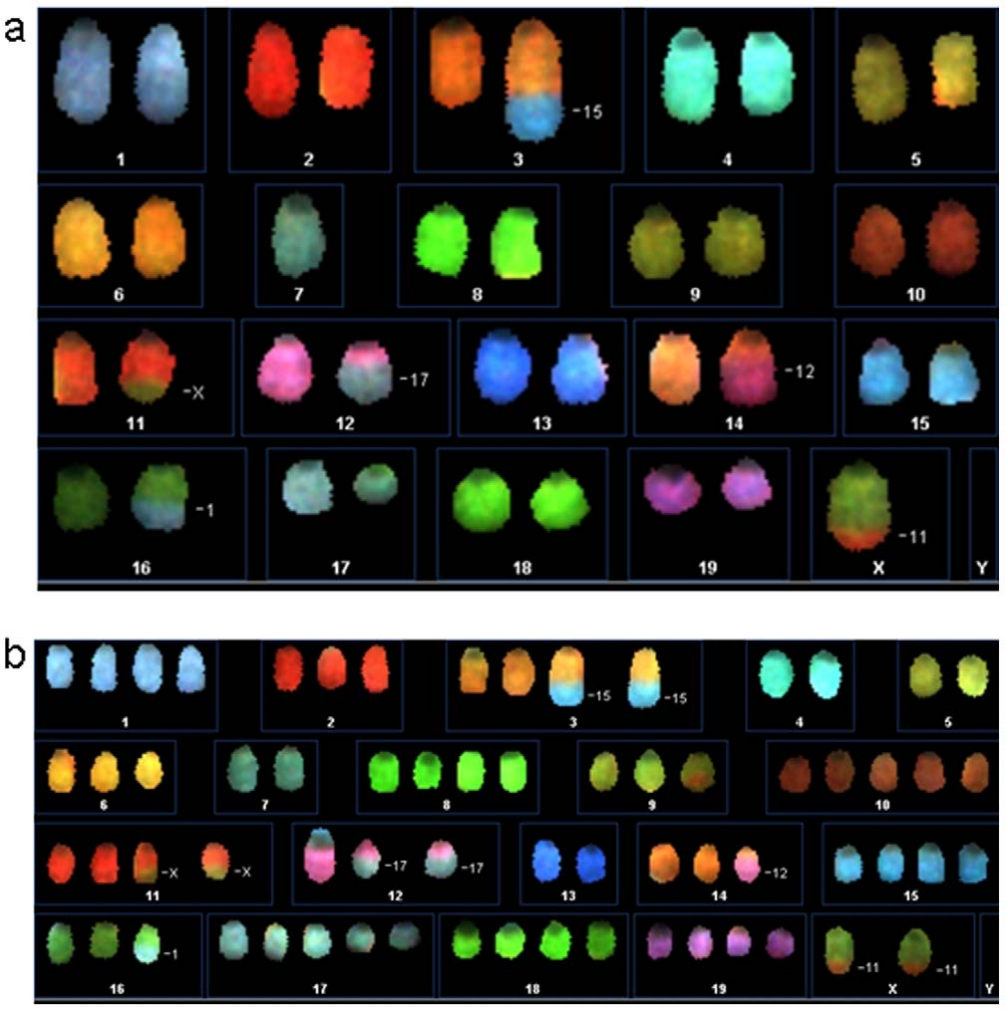
Spectral karyotyping of mMSCs. (a) Shown is the SKY analysis of clone IXH8. SKY analysis revealed clonal structural and numerical chromosomal alterations as demonstrated in the spectral image of a representative diploid metaphase. (b) In most metaphases, we observed a hypertriploid (representative metaphase shown here) to an almost hypotetraploid chromosome complement with loss of the Y-chromosome in all metaphases analyzed.

Stably integrated eGFP-sequences were used for tracing donor cells in recipients after transplantation. Immediately after injection, 20.2%±15.7 of transfused cells could be detected in the peripheral blood (Figure 4a). Within 24 hours, eGFP-positive donor cells were diluted out from PB and trapped in lungs but not in BM, spleen or liver (Figure 4a insert). At day 10 and later on, no donor cells remained in any investigated tissue (PB, thymus, lymph node, liver, spleen, lung, intestine, aorta/vena cava and abdominal fat; not shown). A standard curve for eGFP-BM and assessment of donor cells in long-term survivors (see Materials and Methods) is shown in Figure 4b. This corresponds to results from repeated PB flow cytometry analysis of recipients within the 7-month period where no eGFP-positive or CD45.2 donor cells were found (not shown). Although we transplanted male donor mMSCs into female recipients, the Y-chromosome was not available for chimerism analysis. Among various structural chromosomal alterations and massive aneuploidy we surprisingly detected the loss of the Y-chromosome in clonal mMSCs already during the in vitro expansion period using spectral karyotyping (Figure 5).

### MSCs salvage endogeneous hematopoiesis

**Figure 6:**
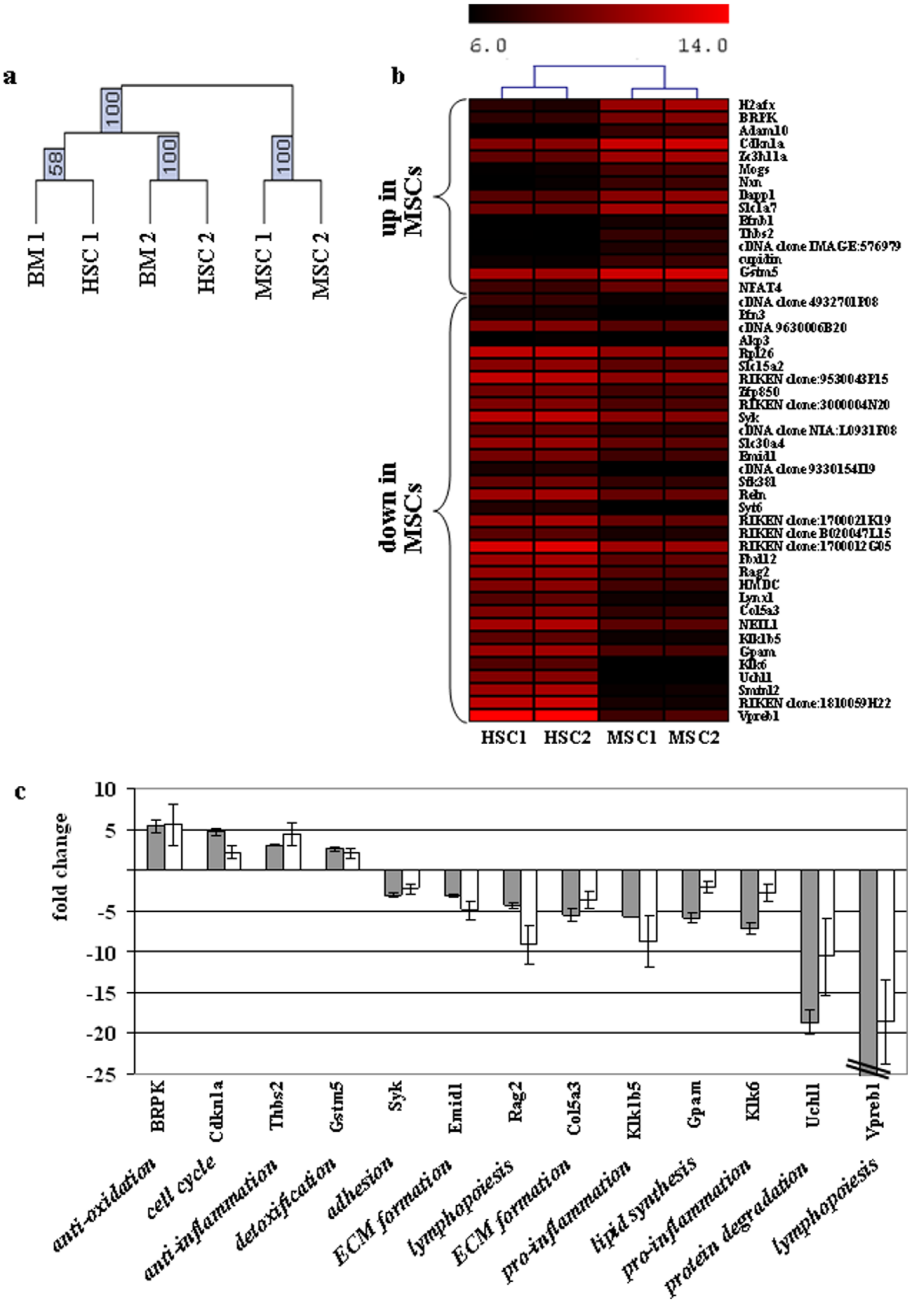
Transplantation of mMSCs leads to gene expression changes in BM supporting rescue of endogenous hematopoiesis. (a) Bootstrap hierarchical clustering of all 20K genes depicted highly stable clusters for HSC/BM vs. MSC. (b) Heat map clustering using average distance and Manhattan metric for 2 microarrays per group hybridized with RNA from pooled BM (HSC: 2 animals/array, MSC: 3 animals/array) is shown for genes summarized in Table 3. (c) Gene expression ratios of selected genes using BM of mice 21 days after transplantation with MSCs (n = 9) or HSCs (n = 10) were investigated for independent cohorts (grey columns: microarray data; white columns: quantitative PCR). Shown are mean ratios ± SEM. P<0.005. Suggested functions of validated genes are shown in italics.

While donor mMSCs did not home to the BM, the gene expression profile in BM changed significantly, clustering as a separate group compared to HSC transplanted mice or age-matched BM (Figure 6a). Impressingly, the clustering of all genes was done without any preselection giving rise to highly stable clusters. A heat map using hierarchical clustering of up- and downregulated genes in the MSC compared to the HSC groups with p≤0.01 and fold changes of ≥2.5 or ≤2.5 illustrates the variations between the samples (Figure 6b). Successful validation of selected genes by quantitative PCR (Table 3) emphasized the beneficial role of mMSCs in endogenous hematopoietic reconstitution (Figure 6c). Transplantation of mMSCs upregulated genes in the BM involved in cell cycle and protection from oxidative stress (Cdkn1a, BRPK) as well as in anti-inflammatory and detoxification processes (Thbs2, Gstm5). In contrast, genes for regulation of proinflammation (Klk6, Klk1b5), protein degradation (Uchl1), adhesion/matrix formation (Sykb, Emid1, Col5a3), lipid synthesis (Gpam), and lymphoid development (Vpreb1, Rag2) were downregulated. The downregulation of genes involved in adhesion and matrix formation particularly suggests lower retention of hematopoietic progenitor cells within the stroma and higher potency to egress into the peripheral circulation.

**Table 3:** Differential gene expression in bone marrow of mice transplanted with HSCs or MSCs.

Accession No	Gene Name	Mean HSC	Mean MSC	ratio	p-value	Suggested functions [references]
**Upregulated in MSCs**						
NM_010436	H2A histone family, member X (H2afx)	7.18	11.05	**14.65**	0.0076	DNA repair [1]
AF316872	protein kinase BRPK/PINK1 (Pten induced putative kinase 1)	7.52	9.94	**5.36**	0.0082	Protection from oxydative stress [2]
NM_007399	a disintegrin and metalloprotease domain 10 (Adam10)	5.69	7.94	**4.74**	0.0097	shedding of inflammation driving substrates [3], [4]
NM_007669	cyclin-dependent kinase inhibitor 1A (P21) (Cdkn1a)	10.19	12.42	**4.68**	0.0031	Cell cycle control [5]
NM_144530	zinc finger CCCH type containing 11A (Zc3h11a)	9.03	11.09	**4.18**	0.0016	Signal transduction [6]
NM_020619	glucosidase 1 (Gcs1), mannosyl-oligosaccharide glucosidase (Mogs)	6.29	8.29	**4.00**	0.0067	Reentry in the mitotic cell cycle, actin cyto-skeletal organization [7]
NM_008750	nucleoredoxin (Nxn)	6.14	7.93	**3.46**	0.0079	accelerated proliferation after oxidative stress [8]
NM_011932	dual adaptor for phosphotyrosine and 3-phosphoinositides 1 (Dapp1), synonym for BAM32	8.73	10.40	**3.18**	0.0055	lymphocyte proliferation [9], [10]
NM_009201	solute carrier family 1, member 7 (Slc1a7)	9.33	11.00	**3.18**	0.0079	Na+-dependent amino acid transporter [11]
NM_010110	ephrin B1 (Efnb1)	5.15	6.82	**3.18**	0.0063	angiogenic remodelling [12]
NM_011581	thrombospondin 2 (Thbs2)	6.02	7.65	**3.10**	0.0020	antiinflammatory [13]
AA116457	mp95d10.r1 Soares_thymus_2NbMT cDNA clone IMAGE:576979 5′	5.63	7.14	**2.85**	0.0089	unknown
AB017136	cupidin/HOMER 2, complete cds	6.36	7.83	**2.77**	0.0029	regulation of T-cell cytokine production [14]
NM_010360	glutathione S-transferase, mu 5 (Gstm5)	11.15	12.54	**2.62**	0.0096	detoxification [15]
D85612	NFATx (NFAT4), complete cds	7.90	9.27	**2.57**	0.0046	Ca^2+^ dependent T-cell activation [16]
**Downregulated in MSCs**						
AV277818	RIKEN full-length enriched, adult male testis (DH5a) cDNA clone 4932701P08 3′,	7.83	6.50	**2.52**	0.0094	unknown
NM_029303	profilin 3 (Pfn3), mRNA	6.67	5.30	**2.58**	0.0051	actin filament assembly (cell motility) [17]
NM_172555	RIKEN cDNA 9630006B20 gene poly(A) polymerase gamma (Papolg)	10.09	8.71	**2.60**	0.0018	Ion channel [18]
NM_007432	alkaline phosphatase 3, intestine, not Mn requiring (Akp3)	6.15	4.74	**2.65**	0.0051	fat absorption [19]
NM_009080	ribosomal protein L26 (Rpl26)	12.03	10.60	**2.70**	0.0030	irradiation-induced apoptosis [20]
NM_021301	solute carrier family 15 (H+/peptide transporter), member 2 (Slc15a2)	10.39	8.95	**2.70**	0.0068	peptide transport [21]
AK020595	adult male urinary bladder cDNA, RIKEN clone:9530043P15 product∶hypothetical Pancreatic ribonuclease	11.94	10.49	**2.73**	0.0098	RNase
BM240956	K0609H05-3 NIA Hematopoietic Stem Cell (Lin−/c-Kit−/Sca-1+) cDNA clone NIA:K060, Zfp850 (zinc finger protein 850)	9.67	8.17	**2.83**	0.0064	unknown
AK013872	12 days embryo head cDNA, RIKEN clone:3000004N20 product∶hypothetical RNA-binding region RNP-1	10.03	8.47	**2.95**	0.0004	unknown
NM_011518	spleen tyrosine kinase (Syk)	11.86	10.29	**2.96**	0.0059	adhesion regulation [22]
BM119878	L0931F08-3 NIA Newborn Kidney cDNA Library (Long) cDNA clone NIA:L0931F08 IMAGE:30003043 3′	9.11	7.52	**3.01**	0.0083	unknown
NM_011774	solute carrier family 30 (zinc transporter), member 4 (Slc30a4)	10.708	9.08	**3.06**	0.0052	enzyme activity [23]
NM_080595	EMI domain containing 1 (Emid1)	9.71	8.09	**3.08**	0.0034	ECM formation [24]
BB078651	RIKEN full-length enriched, adult male diencephalon cDNA clone 9330154I19 3′	6.99	5.365	**3.10**	0.0066	unknown
NM_172734	serine/threonine kinase 38 like (Stk38l)	9.33	7.61	**3.30**	0.0090	neural differentiation [25]
NM_011261	reelin (Reln)	11.03	9.25	**3.44**	0.0041	neural differentiation [26]
NM_018800	synaptotagmin 6 (Syt6)	7.18	5.39	**3.47**	0.0021	vesicular transport proteins [27]
AK006217	adult male testis cDNA, RIKEN clone:1700021K19 product∶hypothetical Serine-rich region/Cysteine-rich	11.07	9.19	**3.70**	0.0099	unknown
BB713538	RIKEN full-length enriched, 2 cells egg cDNA clone B020047L15 3′, mRNA sequence	8.82	6.90	**3.79**	0.0041	unknown
AK005904	adult male testis cDNA, RIKEN clone:1700012G05 product:RIKEN cDNA 1700012G05 gene	12.91	10.95	**3.88**	0.0044	unknown
NM_013911	F-box and leucine-rich repeat protein 12 (Fbxl12)	11.07	9.03	**4.11**	0.0098	ubiquitin-mediated proteolysis [28]
NM_009020	recombination activating gene 2 (Rag2)	10.72	8.61	**4.31**	0.0032	lymphoid development [29]
BC037381	3-hydroxymethyl-3-methylglutaryl-Coenzyme A lyase-like 1 (HMDC)	10.15	7.98	**4.48**	0.0095	phospholipid biosynthesis
NM_011838	Ly6/neurotoxin 1 (Lynx1)	8.81	6.48	**5.01**	0.0091	cell development [30]
NM_016919	collagen, type V, alpha 3 (Col5a3), mRNA	10.12	7.69	**5.39**	0.0072	Structure of fibrillar collagen [31]
AK013322	10, 11 days embryo whole body cDNA, RIKEN clone: 2810450N13 product∶ hypothetical Formamidopyrimidine-DNA glycolase containing protein/ NEIL1	11.33	8.86	**5.53**	0.0041	DNA repair after oxydative damage[32]
NM_008456	kallikrein 1-related peptidase b5 (Klk1b5)	8.95	6.47	**5.58**	0.00006	ECM proteolysis, pro-inflammation [33]
NM_008149	glycerol-3-phosphate acyltransferase, mitochondrial (Gpam)	10.94	8.40	**5.80**	0.0035	glycerolipid synthesis [34]
NM_010639.5	kallikrein 6 (Klk6)	8.67	5.84	**7.13**	0.0029	ECM proteolysis, pro-inflammation [33]
NM_011670	ubiquitin carboxy-terminal hydrolase L1 (Uchl1)	10.15	5.94	**18.52**	0.0008	Protein degradation [35]
BC037993	smoothelin-like 2 (*Smtnl2*)	11.12	6.41	**26.10**	0.0031	Actin stress fibers, inhibition of vasodilation
AK007907	10 day old male pancreas cDNA, RIKEN clone:1810059H22 product∶hypothetical protein	12.27	6.66	**49.02**	0.0024	unknown
NM_016982	pre-B lymphocyte gene 1 (Vpreb1)	14.41	8.25	**71.59**	0.0030	B cell development [36]

Mean gene expression levels from microarray analysis in bone marrow at d21 after transplantation were calculated for 2 arrays per group hybridized with RNA from pooled BM (HSC: 2 animals/array, MSC: 3 animals/array). Selected were genes with p≤0.01 after t-test filtering and ratio of ≥2.5 or ≤2.5. References for suggested functions were selected in the context of cell functionality after transplantation and are shown in Text S1.

## Discussion

While lower dose radiation victims may profit from supportive care, the situation is more serious after irradiation dose higher than 6 Gy. The only curative treatment in these cases is the transplantation of HSCs. However, this alternative depends on whether a suitable donor is available and can be provided quickly. The normal time required to find and deliver a HSC transplant spans over weeks, a frame too long for seriously affected individuals.

**Figure 7:**
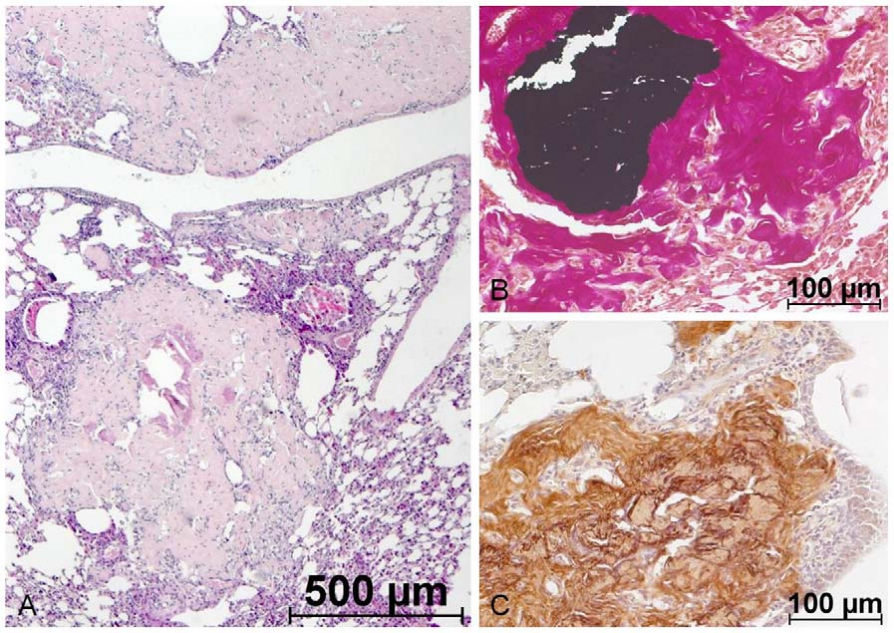
Ectopic ossicles in mice lungs after i.v. transplantation of bulk mMSCs. Mice subjected to total body irradiation and transplanted i.v. with syngeneic MSCs were analyzed after 7 months. In lungs, fibrotic lesions were detected with HE staining (a) which showed the typical dark precipitates in von-Kossa stainings (b) admixed with large Collagen I-positive areas (c) suggesting bone and cartilage containing ossicles.

HSCs reside in close association with osteoblasts and sinusoidal blood vessels within the bone marrow and this association contributes to the maintenance of the HSC pool in vivo. Self-renewal, proliferation and differentiation of HSCs are regulated through intrinsic signals from the BM niche in which the MSCs are a regulatory component [18], [19]. We show here that lethally irradiated mice fully reconstituted the blood system through transplantation of mMSCs with similar kinetics as HSCs. Systemic administration of non-clonal mMSCs resulted in long-term survival of the majority of animals with normal blood cell distribution. Generation and expansion of MSCs from C57BL/6J mice is particularly difficult and time-consuming. Since it is well known that rodent adherent BM cells might contain HSCs over long periods caused by emperiopoiesis [20] among other mechanisms, we formally cannot exclude a contamination of MSC preparations with remaining HSCs. Therefore, clonal mMSCs were evaluated for their reconstitution potential. Here, surprisingly, one population of cloned mMSCs showed an even better reconstituting potential than the bulk population. This clone IXH8 was different from all other MSC cultures: a) morphologically, the clone consisted exclusively of spindle shaped cells indicative for significantly accelerated proliferation of MSCs [21]; that was not observed in bulk and other clonal cultures, and b) flow-cytometrically, we detected increased hematopoietic CD34 and CD45 but no CD105 expression. CD105 has been described as a marker of proliferation and adhesion [22]. We assume that lack of CD105 might increase survival of recipients by lowering lung embolization. All other characteristics corresponded to the ISCT criteria [17], questioning the relevance of CD105 expression particularly for mMSC characterization. Likewise, over-expression of CD105 in cultured endothelial cells has been shown to induce a marked increase in protein levels of inflammatory eNOS [23], suggesting an anti-inflammatory action of CD105-negative mMSCs in our model. Surprisingly, none of the recipients transplanted with clonal mMSCs developed osteosarcomas or fibrotic lesions in lungs as has been observed with non-clonal cultures (Figure 7). Taking together the differences in morphology, antigen expression and survival, we hypothesize that the composition of the expanded mMSCs (e.g. significant amount of nonproliferating osteoprogenitors) rather than the source of the population (mMSC or hMSC as discussed in [24]) causes different outcomes after transplantation.

The survival of recipient animals suggested the homing of mMSC to the BM. We tested this hypothesis using either the Y-chromosome of male donor cells or the stably integrated eGFP as detection marker. Y-chromosome-based chimerism analysis in female recipients could not detect donor cells in any investigated tissue including PB and BM, although animals survived long-term. Spectral karyotyping of clonal mMSCs revealed various structural chromosomal alterations and massive aneuploidy (as did bulk mMSCs) with loss of the Y-chromosome whereas bulk cultures of passage13 were still Y-positive. Hence, mMSCs not only accumulate chromosomal abnormalities during few in vitro passages [24] but also might lose sex-specific chromosomes. Despite this, no tumors or osseous inclusions were formed as shown in Figure 7 and previously described by others [24], [25]. We assume that cloning of mMSCs selected defined populations which do not contain replicative senescent cells as has been regularly detected in bulk populations. The clonal mMSCs might not be prone to stable lung embolization and thus do not lead to eventual tumorous degeneration.

Quantitative PCR for stably integrated eGFP-sequences also failed to detect any donor cells and no eGFP-positive cells were found in PB, BM or thymus by flow-cytometry. These results were unexpected, since forced in vitro differentiation of human MSCs suggested a potential differentiation capability into hematopoietic and endothelial cells, albeit to a rather low degree (Figure S1). Although we cannot completely rule out the presence of single donor cells in the investigated tissues below the detection limit, hematopoietic recovery in recipients due to replenishment with donor cells is unlikely in our setting. Additionally, transplantation of cells with the shown high incidence of chromosomal aberrations would in high probability result in tumor formation. That was not the case in our recipients pointing at limited survival of the cells in vivo. This conclusion contradicts the results of earlier studies analyzing hematopoietic recovery after myeloablative TBI with blood-derived mMSCs [26], [27] and showing donor characteristics in blood and BM. One fundamental difference between both cell sources is the immortalization of cells with SV40 used in these studies, potentially altering BM homing capability of MSCs. Therefore, our results support the concept of impaired transplantability of expanded MSCs [28]–[30] but also challenge the hypothesis of high plasticity of MSCs [31]. Our findings are in contrast to results obtained in immunodeficient mice and monkeys after MSC transfusion [32]–[34]. Francois et al. [32] detected 0.08% to 0.94% of injected human MSCs in several tissues of NOD/SCID mice after TBI+/− local irradiation. In this study, quantitative distribution but no long-term effects of hMSCs were studied. The low percentage might question the physiological relevance of homed hMSCs for intervention in irradiation-induced tissue damage. Recently, the main mechanism through which MSCs counteract global inflammation in sepsis has been attributed to reprogramming of macrophages [35]. This mechanism could be active also in ARS where TNF-α activated MSCs release prostaglandin E2 that acts on the macrophages through the prostaglandin EP2 and EP4 receptors and induces IL-10 secretion. It well might be that, based on this mechanism, the direct injection of hMSCs into the humerus of non-human primates as a single treatment [33] failed to detect any beneficial long-term effect of stromal cells. In an additional approach of ARS in monkey treated with MSC transfusion the one shown animal did not survive 12 days [34]. However, not many of the HSC cotransplanted animals survived long-term either. Because of the low number of experimental animals it remains to be determined if the cells (mean of 14.23% of MSCs were positive for SH2) or the model were not optimal for appropriate assessment. Taken together, our and results from others [36] support the trophic effects of MSCs rather than transdifferentiation.

In contrast to “conventional” MSCs described here, mesodermally derived multipotent adult progenitor cells (MAPCs) with their extraordinarily high plasticity are unable to radioprotect lethally irradiated recipients but possess long-term multilineage hematopoietic repopulating activity, thus preceding HSCs in ontogeny [37], [38]. However, their in vivo equivalent and the true nature (e.g. in vitro artifact) are still unknown. MAPCs seem to differ fundamentally from MSCs in their in vitro and in vivo differentiation potential.

Kinetic analysis of the distribution of eGFP+ donor cells after i.v. transplantation substantiated the fast disappearance from PB. Mouse MSCs trapped in lungs quickly, however without long-term residence and embolization as revealed by lack of donor signals 10 days post-transplant. Additionally, no homing of donor mMSCs to the BM was evident, pointing to salvage of endogenous irradiation-surviving HSCs but not to reconstitution of hematopoiesis by donor MSCs. This conclusion is corroborated by the gene expression profile in BM of MSC-transplanted animals. MSCs in a complex mechanism counteracted factors, e.g. oxidative stress, inflammation and toxification by degradation products which suppress the recovery of remaining HSCs. The MSC-mediated regulation of the niche environment likely is a redundant system that is mediated by several molecules as has been shown for immunoregulation by MSCs [18]. Irradiation produces excessive inflammatory responses [39], contributing to HSC death if left untreated. Among other organs, the lung is especially sensitive towards irradiation damage and may retain MSCs. MSCs interfere with inflammation by changing the gene expression profile not only in the lungs where they dock [40] but also in BM. To do so, MSCs need not necessarily home to the BM but might globally change gene expression or activate the production of systemically counteracting substances. This mechanism has been described for MSCs influencing myocardial infarction [40]. Similarly to mMSCs, injected hMSCs trapped in lungs and affect the production of the anti-inflammatory protein TSG6 which enhances myocardial repair without significant engraftment. Differentiation-independent paracrine MSC-effects also ameliorate acute kidney injury via anti-inflammatory, mitogenic and angiogenic actions [41], [42]. Impressing clinical benefits of MSCs combining surgery and local cellular therapy for the treatment of severe radiation burns has been demonstrated. Uncontrolled clinical symptoms of radiation inflammatory waves successfully could be limited during patient's 8-month follow-up as evidenced by the decrease in the C-reactive protein level observed after each MSC administration thus confirming modulating activity of MSCs in inflammatory processes [43].

Secretion of a broad range of bioactive molecules which alter the tissue microenvironment is now believed to be the main mechanism by which MSCs achieve their therapeutic effect. The transplanted MSCs might, as a principal mechanism, export their inherent trophic effect to unorthodox sites [44]. The outcome is an enhanced regeneration of injured cells, stimulation of proliferation and differentiation of endogenous tissue progenitors, but also a decrease in inflammatory and immune reactions [14].

Our results present the evidence for this highly effective trophic mechanism working also in BM after lethal irradiation in mice. MSCs, moreover, might be helpful in alleviating myelosuppression due to chemotherapy and toxic drug reaction. Whether the results can be translated to humans still has to be shown. Because BM-derived MSCs are easily accessible, can be massively expanded, and stored for prolonged time, they are easily distributed to places in need. We suggest MSC-infusion as an efficient and immediate treatment option after irradiation injuries.

## Materials and Methods

### Mouse MSC generation and characterization

Ethics Statement: Animal experiments were approved by the local ethical committee (License Department Hamburg) under application No. 64/02 and 84/05 and performed according to their guidelines.

Female C57BL/6J-CD45.1 mice (The Jackson Laboratory) represented the recipient population, male C57BL/6J mice with the wild-type CD45 (CD45.2) were used as donor animals.

Mouse MSCs were isolated from male bone marrow and expanded for 9 passages in DMEM/Ham's F12 medium (Biochrom) supplemented with 20% preselected fetal calf serum and 2mM glutamine (both: Invitrogen). The cells were retrovirally labeled with SF91-eGFP [45] at MOI = 3 (bulk population) and expanded or seeded for cloning into ten 96-well plates at 0.3 cells/well. Expanded bulk and selected clonal mMSCs of P14–P18 were characterized according to their differentiation capability into adipo-, chondro- and osteogenic lineages and phenotype as described [46] according to ISCT criteria [17].

Characterization of integration site pattern of clonal mMSCs was carried out as described via LM-PCR [45]. Genomic DNA was digested with Tsp509I giving rise to integration specific fragments of unique lengths. Linker cassettes with known sequences were ligated to the restriction sites. Primer were designed to bind at the known sequences of the provirus and the linker cassette. Amplified PCR products were loaded onto the gel. After gel extraction the bands (red stars) were sequenced. Using the BLAST algorithm, the obtained sequences were aligned to the mouse genome to identify the integration sites.

Chromosome preparation and spectral karyotyping of eGFP-transduced bulk-mMSCs, passage 13 and clone IXH8, passage 20 were performed as described [47].

### Mouse MSC transplantation

TBI was performed using a Cs-137 radiation source. Lethally (9.5 Gy) irradiated female C57BL/6J-CD45.1 mice were i.v. transplanted within 8 hours with 10^6^ cells divided into following groups: **(i)** male bulk (n = 28) or cloned (n = 59; irradiation controls n = 15) mMSCs of P15–P20 for investigation of long-term survival; **(ii)** clone IXH8 mMSCs (n = 32) to investigate the in vivo distribution of donor mMSCs; **(iii)** BM (n = 4, named HSC) or bulk mMSCs (n = 6, named MSC) for microarray analysis; **(iv)** HSC (n = 10) or bulk mMSCs (n = 9) to validate the differential gene expressions obtained with the microarray.

### Sample acquisition and examination

From experimental animals of group **(i)** blood samples were taken retroorbitally and cell counts analyzed using a Coulter Onyx. Seven months later, PB, BM, thymus, lymph node, liver, spleen, lung, intestine, aorta/vena cava and abdominal fat were removed and used for genomic detection of the Y-chromosome (Sry) and/or eGFP via quantitative PCR (Table S2). Parts of lung tissues were fixed in formalin, paraffin embedded and serial cuts stained with HE, von-Kossa stain visualizing calcium precipitates and Collagen I.

Genomic DNA from PB and BM was isolated the same day using innuPREP Blood DNA Mini kit (analytikjena), DNA from all other snap frozen organs with Invisorb Spin Tissue Mini Kit (Invitek). To yield a standard curve, male/GFP-positive DNA was diluted at decreasing concentrations in female/GFP-negative DNA. Quantitative PCR reactions were performed on a Mx3000P (Stratagene). Reaction mixture contained 50 ng of DNA, SYBR Premix Ex Taq (Takara Bio INC), and 200 nM primers (MWG-BIOTECH AG). The threshold cycle (Ct) was determined for each reaction by the MxPro Software. Amplification efficiency was calculated by the sliding window method (LinReg software)[48]. Normalization of expression values was done using *Rps27a* for eGFP and *control chro11* for Y-chromosome tests. Additionally, parts of freshly isolated PB, BM and thymus were investigated for donor derived eGFP- and CD45.2-antigen-expression using flow cytometry as described [45].

Blood samples from animals of group **(ii)** were taken at different time points (20 min, 2, 4, 8, 12 and 24 hours; n = 8 for each time point) after transplantation and isolated DNA used for Sry/eGFP-chromosomal quantitative PCR. At day 1 and 10, PB, BM, lung, liver and spleen were obtained for DNA isolation. All samples were quantitatively tested for a) the Y-chromosome of recipient cells and b) the stably integrated eGFP.

From experimental animals of group **(iii)** at d21, BM from HSC- and mMSC-transplanted groups was flushed combining the cells of 2 or 3 mice respectively and used for RNA isolation using Invisorb Spin Cell RNA Mini Kit (Invitek). Non-manipulated BM of age-matched mice (n = 4, named BM) was used as control. Differential gene expression was investigated using CodeLink UniSet Mouse 20K I Bioarrays as described previously [46]. For each group (BM, HSC, MSC), two arrays were hybridized. Gene expression profiles of all genes were grouped by hierarchical clustering (TIGR MeV v.4.5.1; Manhattan distance, average linkage). All data is MIAME compliant and the raw and processed data has been deposited in a MIAME compliant database at the gene expression omnibus (GEO) under accession GSE21867.

Validation of differential gene expression of genes with a fold change of ≥2.5 was done on the independent mouse group **(iv)** using d21 BM of 10 animals transplanted with HSCs and 9 animals transplanted with clonal IXH8 mMSCs. Primers used are listed in Table S2. Differences in gene expressions in BM of MSC and HSC transplanted animals were determined based on the ΔΔCt method normalized for *Taf12* and *Rps27a*.

### Statistical analysis

For statistical analysis, unpaired and two-tailed Student's t-test was applied. P-values <0.05 were considered statistically significant.

## Supporting Information

Figure S1 In vitro differentiated human MSCs express hematopoietic and endothelial genes and proteins. In vitro differentiated human MSCs express hematopoietic and endothelial genes and proteins. Use of human bone marrow for research was approved by the Ethical Committee of Hamburg. All experiments have been conducted according to the principles of the Declaration of Helsinki. After written informed consent, BM-derived human MSCs (hMSCs) were cloned and expanded in DMEM/LG+10% preselected FCS. Hematopoietic predifferentiation was carried out in serum-containing (HC1) conditions using IMDM+10% FCS+10% horse serum +10−6 M hydrocortisone, mixed 1∶1 with MethoCult H4434 (containing SCF, GM-CSF, IL-3, and Epo) for 2 weeks followed by differentiation for 2 weeks in MethoCult H4435 (containing SCF, GM-CSF, IL-3, IL-6, G-CSF and Epo). A 2-week serum-free (HC2) predifferentiation consisted of StemSpan+SCF+flt3-ligand+TPO+GM-CSF+Epo followed by a 2-week differentiation in MethoCult H4436 (containing SCF, GM-CSF, IL-3, IL-6, G-CSF and Epo). For endothelial differentiation (EC), hMSCs were seeded in fibronectin-coated wells in serum-free StemSpan supplemented with SCF, flt3-ligand, TPO, and Epo for a 2-week prestimulation, followed by a 2-weeks stimulation with StemSpan supplemented with VEGF, FGF-2, IGF, and insulin. Comparative gene expression was tested with qPCR before and after differentiation and normalized to GAPDH and RPS27A. Proteins were detected immunocytochemically on cytospins. (A) Out of 5 clones, IVF1 showed upregulated expression of genes characteristic for early (CD117, CD133, CD41, CD45, EPOR) and mature (CD14, CD16, GlyA, CD31, PDPN) hematopoietic cells as well as several myeloid-lineage transcription factors (GATA1, GATA2, GATA3, RUNX1, NOTCH1, SCL). This upregulation was particularly detected under HC1 (black columns) but also to some extent under HC2 (light grey columns) conditions. Stimulation of endothelial differentiation resulted in gene upregulation of primarily CD31 and KDR (lower part of (a) in dark grey). (B) In concordance with increased gene expression, immunocytochemistry demonstrated expression of early and late hematopoietic (HC1 differentiation shown in the left, HC2 in the middle column) as well as endothelial (EC, right column) antigens as indicated. Either single or, where available, clusters of positive cells are shown. On most cytospins, only single positive cells for the respective antigen were detected. In EC, yellow indicates positivity for both antigens analyzed. All cytospins were counterstained with DAPI to visualize nuclei. The lower part of the right column (“clusters”) shows typical examples of closely clustered dissociation-resistant cells. The most efficient differentiation of hMSCs was obtained when either MSCs (upper picture, white arrows) or already differentiated megakaryocytes (lower picture, white arrow) provided the differentiation environment mimicking the stromal compartment structure in vitro. Human MSCs positive for hematopoietic or endothelial antigens after differentiation decreased cell size from 28.9+/−6.6 to 15.7+/−3.5 µm as well as size and shape of nuclei. Original magnification ×400. Abbreviations: GlyA, glycophorin A; EPOR, erythropoietin receptor; PDPN, podoplanin; VWF, von Willebrand factor; KDR, Kinase insert domain receptor, also referred to as VEGFR2 or FLK1; nd, not detected. Bar: 10µm.
(0.55 MB TIF)

Table S1 Characterization of mMSC clones with LM-PCR. For each clone, the analyzed bands demonstrating the integration sites are shown. Column “position to Transcription Start Site” describes the location of the provirus: “+” means the provirus is located 3′- to the transcriptional start site of the listed gene, “−” means the provirus is located 5′- the transcriptional start site of the listed gene. Adjacent genes of the integration sites were determined in a window of 200kb. Column “orientation” shows the orientation of the provirus with regard to the listed gene.
(0.10 MB DOC)

Table S2 Sequences of primers used for quantitative PCR.
(0.05 MB DOC)

Text S1 Supplementary Text to Table 3. References cited in Table 3 are selected in the context of cell functionality after transplantation.
(0.04 MB DOC)
