# Supplementary figures and images for: Radiation Rescue: Mesenchymal Stromal Cells Protect from Lethal Irradiation
Source: PLoS One. 2011 Jan 5;6(1):e14486. doi: 10.1371/journal.pone.0014486 (PMC3016319; doi:10.1371/journal.pone.0014486)

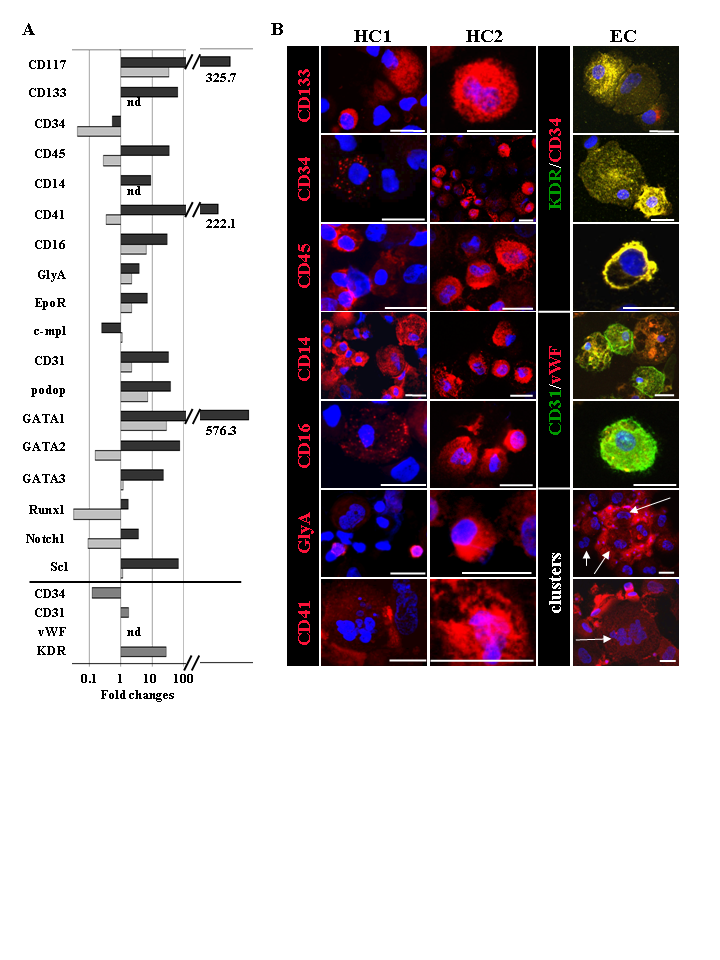

Supplement: Figure S1 — In vitro differentiated human MSCs express hematopoietic and endothelial genes and proteins. In vitro differentiated human MSCs express hematopoietic and endothelial genes and proteins. Use of human bone marrow for research was approved by the Ethical Committee of Hamburg. All experiments have been conducted according to the principles of the Declaration of Helsinki. After written informed consent, BM-derived human MSCs (hMSCs) were cloned and expanded in DMEM/LG+10% preselected FCS. Hematopoietic predifferentiation was carried out in serum-containing (HC1) conditions using IMDM+10% FCS+10% horse serum +10−6 M hydrocortisone, mixed 1∶1 with MethoCult H4434 (containing SCF, GM-CSF, IL-3, and Epo) for 2 weeks followed by differentiation for 2 weeks in MethoCult H4435 (containing SCF, GM-CSF, IL-3, IL-6, G-CSF and Epo). A 2-week serum-free (HC2) predifferentiation consisted of StemSpan+SCF+flt3-ligand+TPO+GM-CSF+Epo followed by a 2-week differentiation in MethoCult H4436 (containing SCF, GM-CSF, IL-3, IL-6, G-CSF and Epo). For endothelial differentiation (EC), hMSCs were seeded in fibronectin-coated wells in serum-free StemSpan supplemented with SCF, flt3-ligand, TPO, and Epo for a 2-week prestimulation, followed by a 2-weeks stimulation with StemSpan supplemented with VEGF, FGF-2, IGF, and insulin. Comparative gene expression was tested with qPCR before and after differentiation and normalized to GAPDH and RPS27A. Proteins were detected immunocytochemically on cytospins. (A) Out of 5 clones, IVF1 showed upregulated expression of genes characteristic for early (CD117, CD133, CD41, CD45, EPOR) and mature (CD14, CD16, GlyA, CD31, PDPN) hematopoietic cells as well as several myeloid-lineage transcription factors (GATA1, GATA2, GATA3, RUNX1, NOTCH1, SCL). This upregulation was particularly detected under HC1 (black columns) but also to some extent under HC2 (light grey columns) conditions. Stimulation of endothelial differentiation resulted in gene upregulation of primaril [file pone.0014486.s001.tif]
