# Supplementary material for: Radiation Rescue: Mesenchymal Stromal Cells Protect from Lethal Irradiation
Source: PLoS One. 2011 Jan 5;6(1):e14486. doi: 10.1371/journal.pone.0014486 (PMC3016319; doi:10.1371/journal.pone.0014486)
Supplement: Table S1 — Characterization of mMSC clones with LM-PCR. For each clone, the analyzed bands demonstrating the integration sites are shown. Column “position to Transcription Start Site” describes the location of the provirus: “+” means the provirus is located 3′- to the transcriptional start site of the listed gene, “−” means the provirus is located 5′- the transcriptional start site of the listed gene. Adjacent genes of the integration sites were determined in a window of 200kb. Column “orientation” shows the orientation of the provirus with regard to the listed gene. (0.10 MB DOC) [file pone.0014486.s002.doc]

Supplementary Table1: Characterization of mMSC clones with LM-PCR.

For each clone, the analyzed bands demonstrating the integration sites are shown. Column “position to Transcription Start Site” describes the location of the provirus: “+” means the provirus is located 3´- to the transcriptional start site of the listed gene, “-“ means the provirus is located 5´- the transcriptional start site of the listed gene. Adjacent genes of the integration sites were determined in a window of 200kb. Column “orientation” shows the orientation of the provirus with regard to the listed gene.

| **mMSC clone** | **seq ID** |  | **locus** | **chromosome** | **(proposed ) function** | **position to Transcription Start Site** | **orientation** |
| --- | --- | --- | --- | --- | --- | --- | --- |
| IV H7 | H7b-1 | 16323 | Inhba | 13 A1 | inhibin beta-A (=growth receptor activity) | + 1 kb  1. intron | F |
|  |  | 665157 | LOC665157 | 13 A1 | similar to ribosomal protein large PO subunit [pseudo] | - 45 kb | R |
|  |  | 625181 | LOC625181 | 13 A1 | similar to ubiquitin-conjugating enzyme E2L 3 [pseudo] | - 30 kb | F |
|  | H7b-2 |  |  |  | not analyzable |  |  |
|  | H7b-3 | 380711 | Garnl4 | 11 B5 | GTPase activating RANGAP domain-like 4 | + 87 kb  2. intron | R |
|  | H7b-4 |  |  |  | same as H7b-3 |  |  |
|  | H7b-5 |  |  |  | not analyzable |  |  |
| V F10 | F10-1 | 11975 | Atp6v0a1 | 11 D | ATPase, H+ transporting, lysosomal V0 subunit A1 | + 50 kb  21. intron | F |
|  |  | 19285 | Ptrf | 11 D | polymerase I and transcript release factor | - 89 kb | R |
|  |  | 27419 | Naglu | 11 D | alpha-N-acetylglucosaminidase (Sanfilippo disease IIIB) | - 10 kb | F |
|  |  | 15485 | Hsd17b1 | 11 D | hydroxysteroid (17-beta) dehydrogenase 1 | - 19 kb | F |
|  |  | 71743 | Coasy | 11 D | Coenzyme A synthase | - 23 kb | F |
|  |  | 21428 | Mlx | 11 D | MAX-like protein X | - 27 kb | F |
|  |  | 19183 | Psmc3ip | 11 D | proteasome (prosome, macropain) 26S subunit, ATPase 3, interacting protein | + 36 kb | R |
|  |  | 67998 | 1300010M03Rik | 11 D | RIKEN cDNA 1300010M03 gene | + 60 kb | R |
|  |  | 103733 | Tubg1 | 11 D | tubulin, gamma 1 | - 60 kb | F |
|  |  | 103768 | Tubg2 | 11 D | tubulin, gamma 2 | - 96 kb | F |
|  | F10-2 |  |  |  | same as F10-1 |  |  |
|  | F10-3 | 193813 | Mcfd2 | 17 E4 | multiple coagulation factor deficiency 2 | + 29 kb | F |
|  |  | 225049 | Ttc7 | 17 E4 | tetratricopeptide repeat domain 7 | - 46 kb | R |
|  | F10-4 | 170749 | Mtmr4 | 11 C | myotubularin related protein 4 | + 0,5 kb 1. intron | R |
|  |  | 18952 | Sept 4 | 11 C | septin 4 | + 12 kb | R |
|  |  | 83560 | Tex14 | 11 C | testis expressed gene 14 | + 106 kb | R |
|  |  | 327992 | Hsf5 | 11 C | heat shock transcription factor family member 5 | - 25 kb | R |
|  | F10-5 |  |  |  | same as F10-4 |  |  |
| VIII E7 | E7-1 |  |  |  | internal control |  |  |
|  | E7-2 | 67991 | Btbd14a | 2 A3 | BTB (POZ) domain containing 14A | + 54 kb  3. intron | F |
|  |  | 109299 | C330006A16Rik |  | RIKEN cDNA C330006A16 gene | + 72 kb | F |
|  |  | 98766 | Ubac1 | 2 A3 | ubiquitin associated domain containing 1 | - 47 kb | F |
|  |  | 665199 | LOC665199 | 2 A3 | similar to growth factor receptor-bound protein 2 | - 73 kb | F |
|  |  | 100040100 | LOC100040100 | 2 A3 | hypothetical protein LOC100040100 | - 84 kb | F |
|  |  | 227634 | Camsap1 | 2 A3 | calmodulin regulated spectrin-associated protein 1 | - 85 kb | F |
| IX C2 | C2-1 | 16211 | Kpnb1 | 11 D | karyopherin (importin) beta 1 | - 3 kb | R |
|  |  | 73174 | Tbkbp1 | 11 D | TBK1 binding protein 1 | - 41 kb | R |
|  |  | 57765 | Tbx21 | 11 D | T-box 21 | - 76 kb | R |
|  |  | 19155 | Npepps | 11 D | aminopeptidase puromycin sensitive | + 88 kb | R |
|  | C2-2 | 109113 | Uhrf2 | 19 C2 | ubiquitin-like, containing PHD and RING finger domains 2 | + 1 kb  1. intron | F |
|  |  | 66745 | Trpd52l3 | 19 C2 | tumor protein D52-like 3 | + 28 kb | F |
|  |  | 77125 | Il33 | 19 C2 | interleukin 33 | + 82 kb | F |
| IX H8 | H8-1 | 76438 | Rftn1 | 17 C | raftlin lipid raft linker 1 | + 171 kb 7. intron | R |
|  |  | 668470 | EG668470 | 17 C | predicted gene, EG668470 | - 92 kb | R |
|  | H8-2 |  |  |  | internal control |  |  |
