# Supplementary material for: Radiation Rescue: Mesenchymal Stromal Cells Protect from Lethal Irradiation
Source: PLoS One. 2011 Jan 5;6(1):e14486. doi: 10.1371/journal.pone.0014486 (PMC3016319; doi:10.1371/journal.pone.0014486)
Supplement: Table S2 — Sequences of primers used for quantitative PCR. (0.05 MB DOC) [file pone.0014486.s003.doc]

Supplementary Table 2. Sequences of primers used for quantitative PCR.

| gene of interest | accession No | primer sequence |
| --- | --- | --- |
| *Control Chr11* | NT_096135.5 | sense: 5´-aaaatggtccaggcaagttctgg-3´  antisense: 5´-tttctcccggtgctctgaatgg-3´ |
| *Sry* | NC_000087.6 | sense: 5´-gttttgggactggtgacaattg-3´  antisense: 5´-gtcttgcctgtatgtgatgg-3´ |
| Rps27a | NM_024277.2 | sense: 5´-ccaggataaggaaggaattcctcctg-3´  antisense: 5´-ccagcaccacattcatcagaagg-3´ |
| pSF91 GFPp |  | sense: 5´-tgcagtgcttcagccgcta-3´  antisense: 5´-ggtgcgctcctggacgtag-3´ |
| Taf12 | NM_025579 | sense: 5´- tgctgcctctgattggccttgg -3´  antisense: 5´- catggagccttgtggcgggg -3´ |
| protein kinase BRPK | AF316872 | sense: 5´- cccacaccctaacatcatcc -3´  antisense: 5´- actgggagtctgctcctcaa -3´ |
| Cdkn1a | NM_007669 | sense: 5´- gccttagccctcactctgtg -3´  antisense: 5´- agggccctaccgtcctacta -3´ |
| Thbs2 | NM_011581 | sense: 5´- gggaccacacaaattgatcc -3´  antisense: 5´- tcccagtaggtctgggtcac -3´ |
| Gstm5 | NM_010360 | sense: 5´- cagttcggtcgcgtcagccc -3´  antisense: 5´- ggatagcatgagccagcccgc -3´ |
| Sykb | NM_011518 | sense: 5´- cctggtgaccaagaccatct -3´  antisense: 5´- tagccaaggctaggaggaca -3´ |
| Emid1 | NM_080595 | sense: 5´- ggttggttccctctgtgaaa -3´  antisense: 5´ - taagcagcctggactcaggt -3´ |
| Rag2 | NM_009020 | sense: 5´- gaagaacgcacactcatcca -3´  antisense: 5´- ctgagtctgaggggcttttg -3´ |
| Col5a3 | NM_016919 | sense: 5´- agggaccaactgggaagagt -3´  antisense: 5´- taaagcagatggagccgagt -3´ |
| Klk1b5 | NM_008456 | sense: 5´- ccaactgggttctcactgct -3´  antisense: 5´- tgatgtcagcaggctttttg -3´ |
| Gpam | NM_008149 | sense: 5´- agcaagtcctgcgctatcat -3´  antisense: 5´- ctcgtgtgggtgattgtgac -3´ |
| Klk1 (Klk6) | NM_010639.5 | sense: 5´- cccacaacctgaggatgact -3´  antisense: 5´- cattaggcaggagcttgagg -3´ |
| Uchl1 | NM_011670 | sense: 5´- gaacgaggccatccaggcgg -3´  antisense: 5´- agggcattcgcccatcgagc -3´ |
| Vpreb1 | NM_016982 | sense: 5´-gggagtgggaaggagaaaag - 3´  antisense: 5´-ctcatagcaacaccgcagaa -3´ |
