# Supplementary material for: Radiation Rescue: Mesenchymal Stromal Cells Protect from Lethal Irradiation
Source: PLoS One. 2011 Jan 5;6(1):e14486. doi: 10.1371/journal.pone.0014486 (PMC3016319; doi:10.1371/journal.pone.0014486)
Supplement: Text S1 — Supplementary Text to Table 3. References cited in Table 3 are selected in the context of cell functionality after transplantation. (0.04 MB DOC) [file pone.0014486.s004.doc]

Supplementary Text to Table 3. References cited in Table 3 are selected in the context of cell functionality after transplantation.

**1.** Economopoulou M, Langer HF, Celeste A, Orlova VV, Choi EY, Ma M, Vassilopoulos A, Callen E, Deng C, Bassing CH, Boehm M, Nussenzweig A, Chavakis T. Histone H2AX is integral to hypoxia-driven neovascularization. Nat Med. 2009;15(5):553-558.

**2.** Henchcliffe C, Beal MF. Mitochondrial biology and oxidative stress in Parkinson disease pathogenesis. Nat Clin Pract Neurol. 2008;4(11):600-609.

**3.** Hikita A, Tanaka N, Yamane S, Ikeda Y, Furukawa H, Tohma S, Suzuki R, Tanaka S, Mitomi H, Fukui N. Involvement of a disintegrin and metalloproteinase 10 and 17 in shedding of tumor necrosis factor-alpha. Biochem Cell Biol. 2009;87(4):581-593.

**4.** Crawford HC, Dempsey PJ, Brown G, Adam L, Moss ML. ADAM10 as a therapeutic target for cancer and inflammation. Curr Pharm Des. 2009;15(20):2288-2299.

**5.** Gartel AL. P21(WAF1/CIP1) may be a tumor suppressor after all. Cancer Biol Ther. 2007;6(8):1171-1172.

**6.** Shu H, Chen S, Bi Q, Mumby M, Brekken DL. Identification of phosphoproteins and their phosphorylation sites in the WEHI-231 B lymphoma cell line.Mol Cell Proteomics. 2004;3(3):279-286.

**7.** Blader IJ, Cope MJ, Jackson TR, Profit AA, Greenwood AF, Drubin DG, Prestwich GD, Theibert AB. GCS1, an Arf guanosine triphosphatase-activating protein in Saccharomyces cerevisiae, is required for normal actin cytoskeletal organization in vivo and stimulates actin polymerization in vitro. Mol Biol Cell. 1999;10(3):581-596.

**8.** Funato Y, Michiue T, Asashima M, Miki H. The thioredoxin-related redox-regulating protein nucleoredoxin inhibits Wnt-beta-catenin signalling through dishevelled. Nat Cell Biol. 2006;8(5):501-508.

**9.** Han A, Saijo K, Mecklenbräuker I, Tarakhovsky A, Nussenzweig MC. Bam32 links the B cell receptor to ERK and JNK and mediates B cell proliferation but not survival.Immunity. 2003;19(4):621-632.

**10.** Sommers CL, Gurson JM, Surana R, Barda-Saad M, Lee J, Kishor A, Li W, Gasser AJ, Barr VA, Miyaji M, Love PE, Samelson LE. Bam32: a novel mediator of Erk activation in T cells. Int Immunol. 2008;20(7):811-818.

**11.** Utsunomiya-Tate N, Endou H, Kanai Y. Cloning and functional characterization of a system ASC-like Na+-dependent neutral amino acid transporter. J Biol Chem. 1996;271(25):14883-14890.

**12.** Zhang J, Hughes S. Role of the ephrin and Eph receptor tyrosine kinase families in angiogenesis and development of the cardiovascular system. J Pathol. 2006;208(4):453-461.

**13.** Park YW, Kang YM, Butterfield J, Detmar M, Goronzy JJ, Weyand CM. Thrombospondin 2 functions as an endogenous regulator of angiogenesis and inflammation in rheumatoid arthritis.Am J Pathol. 2004;165(6):2087-2098.

**14.** Huang GN, Huso DL, Bouyain S, Tu J, McCorkell KA, May MJ, Zhu Y, Lutz M, Collins S, Dehoff M, Kang S, Whartenby K, Powell J, Leahy D, Worley PF. NFAT binding and regulation of T cell activation by the cytoplasmic scaffolding Homer proteins. Science. 2008;319(5862):476-481.

**15.** Wilce MC, Parker MW. Structure and function of glutathione S-transferases. Biochim Biophys Acta. 1994;1205(1):1-18.

**16**. Macian F. NFAT proteins: key regulators of T-cell development and function. Nature Reviews Immunology 2005; 5, 472-484.

**17.** Birbach A. Profilin, a multi-modal regulator of neuronal plasticity. Bioessays. 2008;30(10):994-1002.

**18.** Kahle KT, Rinehart J, Giebisch G, Gamba G, Hebert SC, Lifton RP. A novel protein kinase signaling pathway essential for blood pressure regulation in humans. Trends Endocrinol Metab. 2008;19(3):91-95.

**19.** Nakano T, Inoue I, Koyama I, Kanazawa K, Nakamura K, Narisawa S, Tanaka K, Akita M, Masuyama T, Seo M, Hokari S, Katayama S, Alpers DH, Millán JL, Komoda T. Disruption of the murine intestinal alkaline phosphatase gene Akp3 impairs lipid transcytosis and induces visceral fat accumulation and hepatic steatosis. Am J Physiol Gastrointest Liver Physiol. 2007;292(5):G1439-449.

**20.** Takagi M, Absalon MJ, McLure KG, Kastan MB. Regulation of p53 translation and induction after DNA damage by ribosomal protein L26 and nucleolin. Cell. 2005;123(1):49-63.

**21.** Rubio-Aliaga I, Daniel H. Peptide transporters and their roles in physiological processes and drug disposition. Xenobiotica. 2008;38(7-8):1022-1042.

**22.** Sung YM, Xu X, Sun J, Mueller D, Sentissi K, Johnson P, Urbach E, Seillier-Moiseiwitsch F, Johnson MD, Mueller SC. Tumor suppressor function of Syk in human MCF10A in vitro and normal mouse mammary epithelium in vivo. PLoS One. 2009;4(10):e7445.

**23.** Kambe T, Yamaguchi-Iwai Y, Sasaki R, Nagao M. Overview of mammalian zinc transporters. Cell Mol Life Sci. 2004;61(1):49-68.

**24.** Leimeister C, Steidl C, Schumacher N, Erhard S, Gessler M. Developmental expression and biochemical characterization of Emu family members. Dev Biol. 2002;249(2):204-218.

**25.** Stork O, Zhdanov A, Kudersky A, Yoshikawa T, Obata K, Pape HC. Neuronal functions of the novel serine/threonine kinase Ndr2. J Biol Chem. 2004;279(44):45773-45781.

**26.** Durakoglugil MS, Chen Y, White CL, Kavalali ET, Herz J. Reelin signaling antagonizes beta-amyloid at the synapse. Proc Natl Acad Sci U S A. 2009;106(37):15938-15943.

**27.** Hutt DM, Baltz JM, Ngsee JK. Synaptotagmin VI and VIII and syntaxin 2 are essential for the mouse sperm acrosome reaction. J Biol Chem. 2005;280(21):20197-20203.

**28.** Kipreos ET, Pagano M. The F-box protein family. Genome Biol. 2000;1(5):REVIEWS3002.

**29.** Oettinger MA. Activation of V(D)J recombination by RAG1 and RAG2. Trends Genet. 1992;8(12):413-416.

**30.** Hoogduijn MJ, Cheng A, Genever PG. Functional Nicotinic and Muscarinic Receptors on Mesenchymal Stem Cells. Stem Cells Dev. 2009, 18(1): 103-112.

**31.** Gopalakrishnan B, Wang WM, Greenspan DS. Biosynthetic processing of the Pro-alpha1(V)Pro-alpha2(V)Pro-alpha3(V) procollagen heterotrimer. J Biol Chem. 2004;279(29):30904-30912.

**32.** Mori H, Ouchida R, Hijikata A, Kitamura H, Ohara O, Li Y, Gao X, Yasui A, Lloyd RS, Wang JY. Deficiency of the oxidative damage-specific DNA glycosylase NEIL1 leads to reduced germinal center B cell expansion. DNA Repair (Amst). 2009;8(11):1328-1332.

**33.** Eissa A, Diamandis EP. Human tissue kallikreins as promiscuous modulators of homeostatic skin barrier functions. Biol Chem. 2008;389(6):669-680.

**34.** Lewin TM, Schwerbrock NM, Lee DP, Coleman RA. Identification of a new glycerol-3-phosphate acyltransferase isoenzyme, mtGPAT2, in mitochondria. J Biol Chem. 2004;279(14):13488-13495.

**35.** Bheda A, Yue W, Gullapalli A, Whitehurst C, Liu R, Pagano JS, Shackelford J. Positive reciprocal regulation of ubiquitin C-terminal hydrolase L1 and beta-catenin/TCF signaling. PLoS One. 2009;4(6):e5955.

**36.** Mundt C, Licence S, Maxwell G, Melchers F, Mårtensson IL. Only VpreB1, but not VpreB2, is expressed at levels which allow normal development of B cells. Int Immunol. 2006;18(1):163-172.
